# High-Performance Vertical Light-Emitting Transistors Based on ZnO Transistor/Quantum-Dot Light-Emitting Diode Integration and Electron Injection Layer Modification

**DOI:** 10.3390/mi14101933

**Published:** 2023-10-15

**Authors:** Jui-Fen Chang, Jia-Min Yu

**Affiliations:** Department of Optics and Photonics, National Central University, Zhongli 320317, Taiwan; link62260@gmail.com

**Keywords:** inorganic transistors, quantum-dot light-emitting diodes, vertical light-emitting transistors

## Abstract

Vertical light-emitting transistors (VLETs) consisting of vertically stacked unipolar transistors and organic light-emitting diodes (OLEDs) have been proposed as a prospective building block for display technologies. In addition to OLEDs, quantum-dot (QD) LEDs (QLEDs) with high brightness and high color purity have also become attractive light-emitting devices for display applications. However, few studies have attempted to integrate QLEDs into VLETs, as this not only involves technical issues such as compatible solution process of QDs and fine patterning of electrodes in multilayer stacked geometries but also requires a high driving current that is demanding on transistor design. Here we show that these integration issues of QLEDs can be addressed by using inorganic transistors with robust processability and high mobility, such as the studied ZnO transistor, which facilitates simple fabrication of QD VLETs (QVLETs) with efficient emission in the patterned channel area, suitable for high-resolution display applications. We perform a detailed optimization of QVLET by modifying ZnO:polyethylenimine nanocomposite as the electron injection layer (EIL) between the integrated ZnO transistor/QLED, and achieve the highest external quantum efficiency of ~3% and uniform emission in the patterned transistor channel. Furthermore, combined with a systematic study of corresponding QLEDs, electron-only diodes, and electroluminescence images, we provide a deeper understanding of the effect of EIL modification on current balance and distribution, and thus on QVLET performance.

## 1. Introduction

In recent years, vertical light-emitting transistors (VLETs) have gained extensive attention due to their potential applications for display technologies [1,2,3,4]. VLETs are generally developed based on the vertical stacking of unipolar organic transistors and organic light-emitting diodes (OLEDs), with the source electrode sandwiched between the outer gate and drain electrodes. This vertical architecture with a short channel length (typically sub-micron) offers important advantages in terms of low operating voltage, high switching frequency, and large aperture ratio, and also allows flexible combinations of different functional materials. The operation of VLETs relies on the interaction between the vertical gate-source and drain-source fields, so one carrier type injected from the source can be modulated by the gate-source field and pulled toward the LED emitting layer by the drain-source field to recombine with another carrier type injected from the drain. Two transistor geometries with different source designs for gate modulation, namely the Schottky barrier transistor and encapsulated source electrode transistor, have been adopted to construct VLETs. Schottky barrier transistors employ an appropriate form of source electrode with transparency to DC electric fields, such as carbon-based and perforated conductors, on dielectric to avoid shielding the gate field [5,6,7,8,9]. Their switching performance depends critically on the design of the Schottky barrier and perforated source pattern [10,11,12]. However, these source electrodes usually involve complex manufacturing processes with poor regularity, which is not conducive to cost-effectiveness, large-area production, and current control. In another VLET geometry, the source electrode is embedded in the active layer and covered with an insulator, enabling gate-modulated current injection from the bottom source surface into the underlying active layer in a manner similar to conventional planar top-contact transistors [13,14,15,16,17]. This geometry is advantageous to avoid the gate-shielding effect and block non-gate leakage, so the on- and off-current can be effectively controlled via a simple source pattern to achieve large switching ratios [17,18], and the injection barrier can also be lowered to enhance on-current [19,20]. The main challenge, however, lies in the fine patterning of the encapsulated source electrodes without damaging the underlying semiconductor layer, which is particularly problematic for organic semiconductors that are incompatible with standard lithographic processes.

Based on the encapsulated source electrode transistor geometry, we recently reported inorganic/organic hybrid VLETs with a bottom-up integration of ZnO transistors and OLEDs [21]. The advantages of using inorganic transistors instead of organic counterparts in VLETs are manifold. From a technical perspective, inorganic transistors are compatible with standard photolithography processes and resistant to most organic solvents, which can simplify the fabrication of VLETs of various sizes and integrate with a variety of solution-processed and evaporated LEDs. Moreover, inorganic transistors with ohmic injection and high mobility can generate extremely high currents, capable of driving general OLEDs and even other devices that require high operating currents, such as quantum-dot (QD) LEDs (QLEDs). QLEDs featuring high brightness, pure color, and solution processability have emerged as attractive kinds of electroluminescent devices favored by low-cost, large-area display and lighting technologies [22,23,24,25]. However, to date, there have been few studies on QD-VLETs (QVLETs), possibly due to the technical issues involved in solution deposition of QDs and fine patterning of electrodes in multilayer stacked geometries, as well as the rigorous demand for transistor design to achieve high driving current. Previously, Chen et al. proposed a unique design of QVLETs based on a top-gate Schottky barrier transistor geometry, where an organic transistor with a metallic nanowire network source electrode was deposited on the QD emitting layer via compatible solution processes [26]. Although such QVLETs achieved high luminescent efficiencies driven by high-mobility organic transistors, difficulties remained in precisely patterning the perforated source and top gate above the organic channel to control current output and emission area, which are the typical issues of Schottky barrier transistors for applications. Following our recent study, herein, we develop QVLETs by integrating high-mobility ZnO transistors and solution-processed QLEDs based on the encapsulated source electrode transistor geometry. We show that the technical advantages and excellent electrical properties of ZnO transistors enable easy fabrication of QVLETs and achieve emission performance close to that of pristine QLEDs as well as controlled emission area in the patterned channel, benefiting high-resolution display applications. To optimize QVLET, we investigate the modification of ZnO and polyethylenimine (ZnO:PEI) nanocomposite as the electron injection layer (EIL) between the integrated ZnO transistor and QLED. We find that blending an appropriate ratio of PEI in EIL can significantly improve the emission efficiency and emission uniformity in the channel area compared to the case without PEI. The effect of EIL modification on current balance and distribution that accounts for the improved device performance is also discussed.

## 2. Device Fabrication and Characterization Method

**Figure 1:**
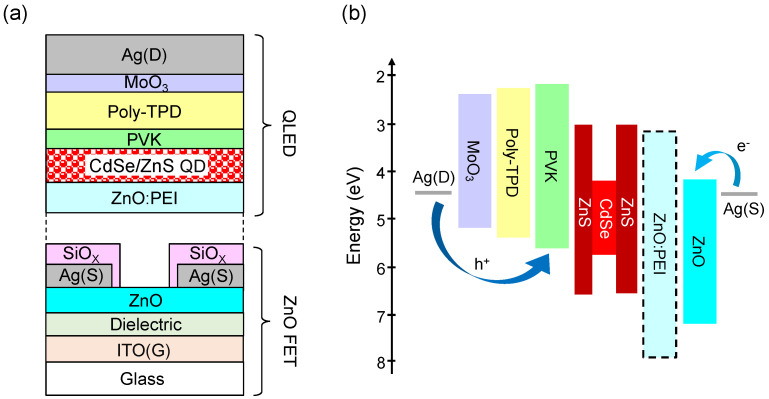
(**a**) Schematic of QVLET based on a vertical integration of ZnO field-effect transistor (FET) and QLED. (**b**) Energy level diagram of the used materials in the QVLET.

Figure 1a illustrates the QVLET architecture, where the QLED is stacked on the ZnO field-effect transistor with the insulator-encapsulated source electrode to confine the current path from the bottom source surface to the top drain. The fabrication of the bottom ZnO transistor was detailed in our previous study [21]. Briefly, a 150 nm indium tin oxide (ITO) pattern on a glass substrate was used as the gate electrode. The gate dielectric (15 nm Al_2_O_3_/15 nm HfO_2_ bilayer) and a 30 nm ZnO layer were deposited using the atomic layer deposition (ALD) technique, which yields a low gate leakage, a high areal capacitance of 350 nF/cm^2^, and a high electron mobility of ~10 cm^2^/Vs for the ZnO transistor (see transfer characteristics in Supplementary Materials, Figure S1). The high transistor mobility is important to support lateral electron transport away from source injection in the QVLET, forming a long-range electron density gradient (channel depth) to produce strong surface emission. We patterned the ALD-ZnO layer into a rectangle with area *A* = 500 × 1400 μm^2^ to define the channel area for driving the top integrated QLED. The source electrode (40 nm silver, Ag) encapsulated with 250 nm silicon oxide (SiO_X_) was deposited using thermal evaporation and patterned using a self-aligned photolithography process. Silver electrodes deposited on ZnO can form ohmic contacts for efficient current injection. The source design on the ZnO pattern also plays a geometrical role in controlling the current output and distribution and affects the intensity and spatial uniformity of QVLET emission. In order to inject high current and consider the channel depth that ZnO mobility can support, we designed an interdigitated source pattern with 11 periodic stripes on the ZnO pattern, each stripe width of 10 μm, and the bare aperture width of 100 μm between two adjacent stripes, corresponding to a geometrical aperture ratio (the ratio of the total source aperture area to the ZnO pattern) of up to 92%. As shown later, this simple source design allows us to achieve bright and uniform emission over the ZnO pattern in the optimized QVLET. Note that in this work, we consider the total source aperture area on ZnO as the effective channel to evaluate the current density for all QVLETs. The top QLED was then constructed with red-emitting CdSe/ZnS core-shell type QD in an inverted configuration. As the energy diagram shown in Figure 1b, CdSe/ZnS QD has relatively low HOMO and LUMO levels, thus requiring appropriate injection/transporting layers to improve electron-hole recombination. We used a 25 nm ZnO:PEI nanocomposite as the EIL deposited on the bottom ZnO transistor [27,28]. The ZnO:PEI nanocomposite was prepared by dissolving zinc acetylacetonate hydrate and PEI in ethanol at a controlled concentration, followed by spin-coating and annealing at 120 °C for 90 min in the ambient environment. Since PEI is a large bandgap insulating polymer containing aliphatic amine groups, blending PEI with ZnO can form a hybrid material that not only has amine oxide dipoles to facilitate electron injection but also increases insulation [29,30], allowing for regulation of electron and hole currents. Here we investigate a series of EILs with ZnO:PEI weight ratios of 1:0.17, 1:0.33, and 1:0.5 for QVLET optimization and compare them to the case without PEI. The remainder of device fabrication was performed in a nitrogen glove box connected to an evaporation chamber. A 25 nm CdSe/ZnS QD film was spin-coated from toluene at a concentration of 15 mg/mL and annealed at 75 °C for 30 min. Subsequently, a 5 nm Poly(9-vinylcarbazole) (PVK) film and a 65 nm Poly[N,N′-bis(4-butylphenyl)-N,N′-bis(phenyl)-benzidine] (Poly-TPD) film were deposited as the electron blocking layer and hole transporting layer, respectively. PVK film was spin-coated from chlorobenzene at a concentration of 5 mg/mL and annealed at 120 °C for 30 min, whereas the Poly-TPD film was spin-coated from toluene at a concentration of 13 mg/mL and annealed at 120 °C for 30 min. Finally, 40 nm MoO_3_ and 120 nm Ag were thermally evaporated as the hole injection layer and anode, respectively. The Ag anode covers the patterned ZnO channel and serves as the drain electrode and reflective mirror of the QVLET.

All the optoelectronic characterizations were measured in a nitrogen glove box using a Keysight B1500A semiconductor parameter analyzer (Santa Rosa, CA, USA). The luminance was measured using a Konica Minolta CS-2000 spectroradiometer (Osaka, Japan), and the light-emission intensity was measured using a Hamamatsu S1227-1010BQ silicon photodiode (Hamamatsu, Japan) placed beneath the device. The electroluminescence (EL) spectrum was collected using an Ocean Optics HR4000 spectrometer (Orlando, FL, USA) with a multimode optical fiber. The EL image was recorded using an optical microscope equipped with a digital camera. The external quantum efficiency (EQE) was calculated from the *J*-*V* characteristic, light-emission intensity, and normalized EL spectrum. The ratio of the current density *J* and the elementary charge gives the number of electrons per second *N*_e_. On the other hand, the light-emission intensity measured by a silicon photodiode represents the wavelength-integrated detected light power. To calculate the total number of emitted photons per second *N*_ph_, the light-emission intensity is divided by the mean photon energy (weighted by the normalized EL spectrum). Thus, EQE = *N*_ph_/*N*_e_ can be obtained.

## 3. Results and Discussion

**Figure 2:**
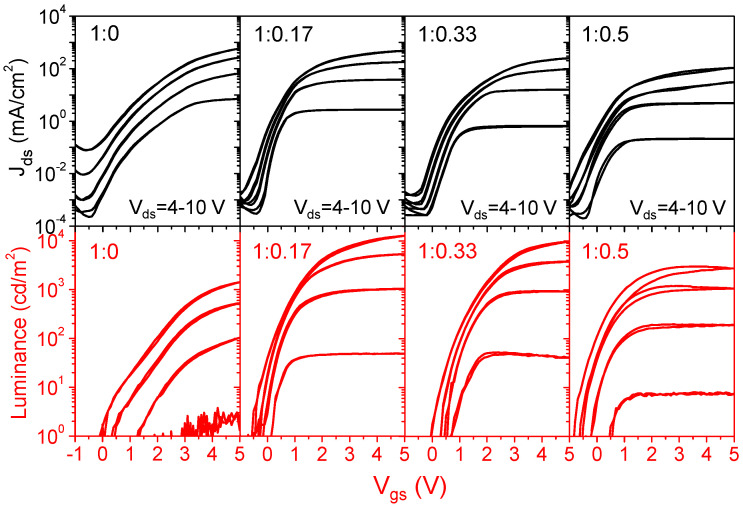
Transfer characteristics of QVLETs with various ZnO:PEI ratios in EIL. *V*_ds_ is varied from 4 to 10 V in 2 V steps.

**Figure 3:**
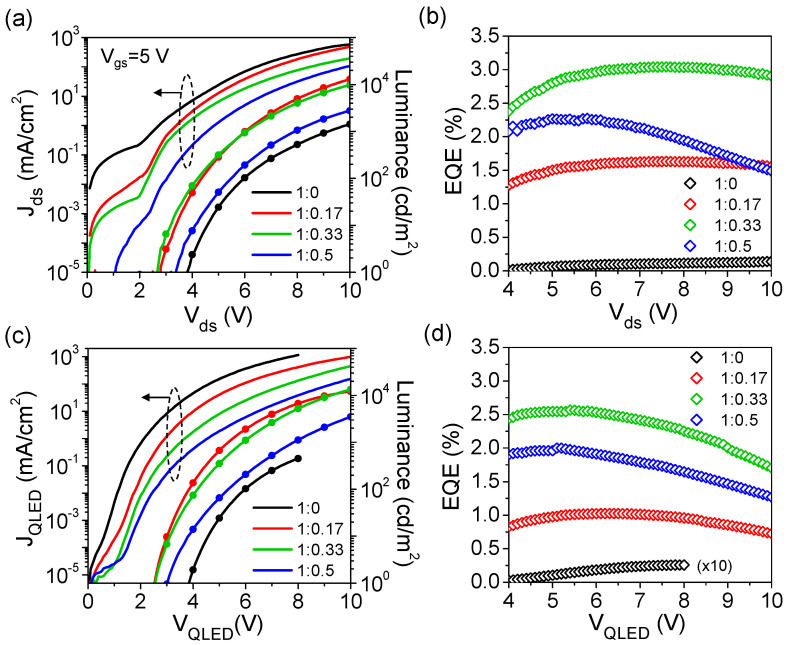
(**a**) Output characteristics and (**b**) EQEs of QVLETs with various ZnO:PEI ratios in EIL at *V*_gs_ = 5 V. (**c**) *J*-*V*-*L* characteristics and (**d**) EQEs of corresponding QLEDs with ITO cathode.

Figure 2 shows the transfer characteristics of the QVLETs performed at *V*_ds_ = 4–10 V for a series of ZnO:PEI ratios in EIL. Overall, these QVLETs driven by the well-designed ZnO transistor exhibit n-type transistor behavior with a near-zero onset voltage, little hysteresis, and low off-current densities suppressed by the SiO_X_ source encapsulation. The effect of varying PEI ratios is mainly observed in the ON state. Among them, the device without PEI in EIL has the highest on-current density but the lowest luminance (570 mA/cm^2^ and 1410 cd/m^2^ at *V*_gs_ = 5 V, *V*_ds_ = 10 V). As the PEI ratio increases, the on-current density tends to decrease, whereas the luminance reveals a nonmonotonic variation—increasing sharply to the highest value at a low ratio of 0.17 (12,740 cd/m^2^ at *V*_gs_ = 5 V, *V*_ds_ = 10 V) and then decreasing slightly at higher ratios. Figure 3a,b further show the output characteristics and extracted EQEs of different devices operating at *V*_gs_ = 5 V. It is clear that the device without PEI in EIL enables current injection at a low voltage but starts emitting at a relatively high turn-on voltage of *V*_ds_ = 3.5–4 V (defined as the voltage at which luminance exceeds 1 cd/m^2^), thus having the lowest luminance and lowest EQE of 0.14%. By contrast, blending PEI in EIL tends to reduce on-current but lower turn-on voltage and significantly improve luminance and EQE. This suggests that an appropriate ratio of PEI in EIL can effectively enhance electron-hole recombination, which may be a consequence of the combined effect of dipole formation and increased insulation to regulate current injection and blocking, as will be discussed in detail later. More specifically, as the PEI ratio varies from 0.17 to 0.5, the luminance seems to decrease with the reduced on-current, whereas the EQE shows an increasing-then-decreasing trend. Overall, the device with a PEI ratio of 0.33 achieves the best performance with the highest EQE of 3.04%, and its luminance is only slightly lower than that of the device with a PEI ratio of 0.17. When the PEI ratio is higher than 0.33, the luminance decreases rapidly with the on-current, causing the turn-on voltage to increase and the EQE to decrease accordingly. Figure 3c,d shows the *J*-*V*-*L* characteristics and extracted EQEs of the corresponding QLEDs with ITO cathode for comparison. It can be seen that despite the different electron injection operations, QVLETs at ON state, and QLEDs exhibit similar characteristics and dependence on the PEI ratio. Table 1 summarizes the electrical and optical parameters of QVLETs and QLEDs. Under the same EIL modification, QVLETs typically have slightly lower current and luminance than QLEDs, which may be due to the internal resistance or hole-blocking property of the ZnO transistor. However, QVLET can even have a higher EQE than QLED. This comparison suggests that the ZnO transistor does not cause significant power consumption that degrades QVLET performance and can even regulate the current balance in conjunction with EIL to achieve better efficiency than QLED.

Supplementary Materials Figure S2 shows a typical EL spectrum of QVLET without PEI in EIL. Basically, the spectra of QVLETs and QLEDs are almost identical and little depend on EIL modification, all revealing narrowband emission with a peak wavelength of approximately 626 nm.

**Figure 4:**
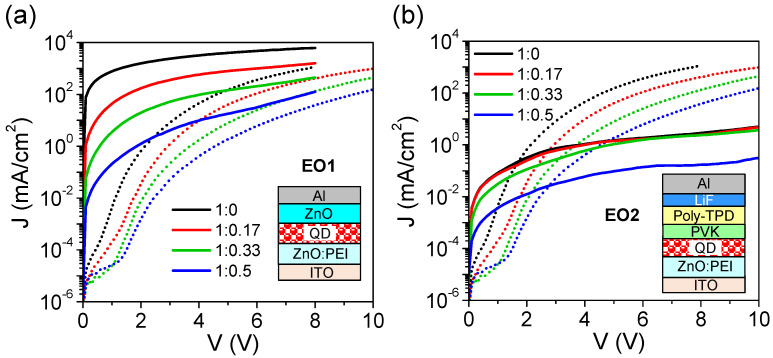
*J-V* characteristics of two types of electron-only devices: (**a**) EO1 and (**b**) EO2 with various ZnO:PEI ratios in EIL. *J-V* characteristics of corresponding QLEDs (dotted lines) are also shown for comparison.

To further understand the effect of blended PEI in EIL on device performance, we characterize two types of electron-only diodes, ITO (150 nm)/ZnO:PEI (25 nm)/QD (25 nm)/ZnO (40 nm)/Al (100 nm) and ITO (150 nm)/ZnO:PEI (25 nm)/QD (25 nm)/PVK (5 nm)/Poly-TPD (65 nm)/LiF (1 nm)/Al (100 nm), denoted as EO1 and EO2, respectively. Figure 4a,b shows the EO1 and EO2 device structures and their *J*-*V* curves with different ZnO:PEI ratios in EIL compared to the corresponding QLEDs. The EO1 device without PVK/Poly-TPD layers was prepared by spin-coating a 40 nm ZnO nanoparticle film with a high work function of 4.3 eV and high conductivity on the QD film, and thus the device resistance mainly results from the EIL and QD film. All the EO1 devices exhibit near-ohmic injection and a rapid increase in current density to high levels beyond QLEDs, indicating that vertical electron transport along the EIL and QD film is very efficient with a small barrier in between. As verified by ultraviolet photoemission spectroscopy measurements, the injection barrier from PEI-free EIL to QD film is estimated to be only 0.09 eV and further decreases with increasing PEI ratio (see discussion in Supplementary Materials, Figures S3 and S4). However, the current density in the EO1 devices is shown to decrease significantly as the PEI ratio increases. These results may suggest that PEI in EIL has a stronger effect in increasing insulation than forming dipoles that facilitate electron injection, resulting in an overall decrease in electron current. On the other hand, the EO2 device with the presence of PVK/Poly-TPD layers is structurally similar to QLED but uses an Al top electrode and a LiF interfacial layer to avoid hole injection, and thus may reveal the electron current in QLED. Also, since we are unable to measure the hole current in the presence of ZnO:PEI EIL, the hole current in QLED can be assessed through the difference between EO2 and QLED. It can be seen that all the EO2 devices still exhibit near-ohmic injection, but the current density is several orders of magnitude lower than that of the EO1 devices and increases slowly with voltage, indicating that electron current in QLED is effectively blocked or depressed by PVK/Poly-TPD layers. Interestingly, compared to the corresponding QLEDs, the EO2 devices have orders of magnitude higher current at low voltages but orders of magnitude lower current at high voltages. From this result, we speculate that the hole current in QLED at a sufficiently high voltage may overcome a large injection barrier from the PVK layer into QD film and exceed the electron current to become the dominant current in QLED. The electron current in the EO2 devices also tends to decrease with increasing PEI ratio, similar to the current variation in the EO1 devices and QLEDs. Apparently, PEI increases the insulation of EIL which imposes a blocking effect on both electron and hole currents, thereby promoting the current balance and recombination in QDs under optimized conditions. This is believed to be the main reason for the enhanced EQE of QVLET and QLED at appropriate PEI ratios. We have also investigated the control LEDs without QD film, as the results shown in Figure S5. In this case, the device without PEI does not emit light, whereas the devices with PEI in EIL show improved emission with EQEs of 10^−3^–10^−2^% for different PEI ratios. Thus, for QVLETs and QLEDs, PEI in EIL may also help inject the small number of electrons leaking through the voids between QDs into the top PVK/Poly-TPD layers, where excitons can be formed and undergo Förster resonance energy transfer to enhance QD emission [31].

**Figure 5:**
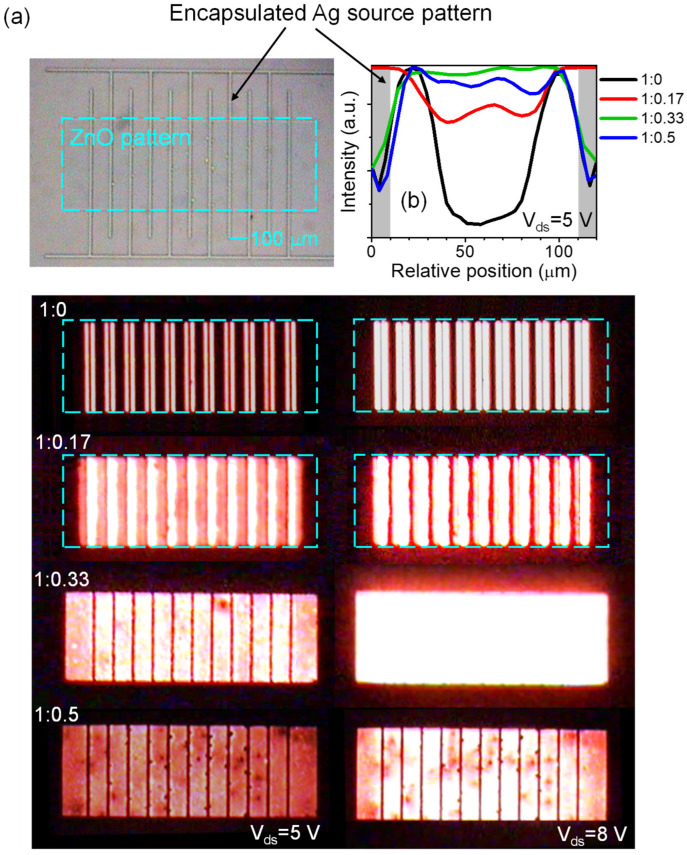
(**a**) EL images of QVLETs with various ZnO:PEI ratios in EIL, recorded during the output scan at *V*_gs_ = 5 V. (**b**) EL intensity distribution of QVLETs with different ZnO:PEI ratios in EIL between two adjacent encapsulated source electrodes in the central region of the ZnO pattern. The intensity is analyzed at *V*_ds_ = 5 V in (**a**) and normalized for all devices to highlight emission uniformity across the source aperture.

Figure 5a shows the EL images of QVLETs with various ZnO:PEI ratios in EIL, recorded during the output scan at *V*_gs_ = 5 V. It can be seen that the QVLET without PEI exhibits a narrow emission zone near the edge of the encapsulated source stripe. Increasing the PEI ratio in EIL appears to broaden the emission zone, making the emission more uniform across the source aperture and spatially matching the ZnO channel area. To better resolve the emission distribution of all devices, we normalized their respective emission intensities at *V*_ds_ = 5 V within the source aperture in the central region of the ZnO pattern, as shown in Figure 5b. Note that the device with the PEI ratio of 0.33 not only has the highest EQE but also the most uniform emission. This optimized device can produce an intense glow at a high bias (*V*_ds_ = 8 V), even eliminating the shadow of source stripes and appearing to emit light from the entire ZnO area. For the higher PEI ratio of 0.5, the device shows less uniform emission with some non-emissive defects, which may reflect a certain degree of inhomogeneity when blending excess PEI with ZnO and is somewhat correlated with a decrease in EQE. Importantly, these EL images provide a clear insight into the lateral electron distribution in the QVLET. As suggested by the previous study [32], the conductive channel in this VLET geometry results from a complex balance between lateral and vertical charge transport in the multilayer stack, which may depend on the mobility and relative energy levels of the stacked materials, and form a channel depth manifested in the length scale of emission zone. Since the electron mobility of the ZnO transistor is several orders of magnitude higher than that of top integrated QLED, and the injection barrier between EIL and QD film is very small, lateral electron transport may take place mainly in the ZnO transistor and be influenced by the conductivity of EIL. We speculate that EIL without PEI has relatively high conductivity for vertical transport (see discussion in Figure 4a), so electrons leaving the source edge can be readily injected into the EIL and QD film under vertical drain field extraction, forming a narrow emission zone close to the source edge. Increasing the PEI ratio in EIL would reduce the conductivity, making it more difficult for electrons to be injected from the ZnO transistor. In this circumstance, electrons tend to diffuse laterally over a longer distance from the source edge before being injected into the EIL, thus broadening the emission zone and smearing out the vertical drain field extraction to some extent. As evident in the devices with high PEI ratios, the emission zone can extend over a hundred micrometers from the source edge without significant variations with *V*_ds_. Taking advantage of such a wide emission zone, the source design on the ZnO pattern can be simplified to achieve uniform emission under different bias conditions, greatly benefiting high-resolution display applications.

## 4. Conclusions

In summary, we develop the high-performance QVLET based on the integration of ZnO transistors and QLEDs and demonstrate the importance of EIL between the two for device optimization. The use of ZnO:PEI nanocomposite as EIL allows the tuning of injection and insulating properties to balance electron and hole currents for effective recombination in the studied QVLETs. By blending an appropriate PEI ratio in EIL, the optimized device can achieve a maximum EQE of ~3% and uniform emission over the defined ZnO transistor channel area, which is a significant improvement compared to a low EQE and narrow emission zone in the device without PEI. This enhanced EQE is similarly observed in the corresponding QLEDs and can be mainly attributed to the PEI increasing the insulation of the EIL, resulting in a current blocking effect and thus improving current balance and recombination. Exploring more efficient QD emitters and further optimizing the injection/transporting layers of stacked QLEDs may lead to higher EQEs for QVLETs. On the other hand, the reduced conductivity of EIL with blended PEI also forces electrons to move laterally over a longer distance in the ZnO transistor before entering the EIL, leading to a broadened emission zone and thus enabling a simple source design to achieve uniform, spatially defined emission in the optimized QVLET. We believe that the studied transistor geometry and nanocomposite EIL can be applied to integrate various QLEDs and allow further improvement of emission property and control of emission area, paving the way for realizing bright, high-resolution QD-based active displays.

## Figures and Tables

**Table 1:** Summary of characteristics of QVLETs at *V*_gs_ = 5 V and corresponding QLEDs with various ZnO:PEI ratios in EIL. Since QLED without PEI is prone to breakdown when operating at voltages higher than 8 V, the current density and luminance at 8 V are listed for all devices to allow for a fair comparison.

	QVLET/QLED		
ZnO:PEI	J@8 V (mA/cm^2^)	Lum@8 V (cd/m^2^)	Max. EQE (%)
1:0	268/1149	528/452	0.14/0.025
1:0.17	183/413	4969/6707	1.63/1.02
1:0.33	77/134	4033/5252	3.04/2.57
1:0.5	31/39	1064/1124	2.26/2.02

## Data Availability

Data sharing not applicable.

## Supplementary Materials

The following supporting information can be downloaded at: https://www.mdpi.com/article/10.3390/mi14101933/s1, Figure S1: transfer characteristics of ZnO transistor; Figure S2: EL spectrum of QVLET without PEI in EIL; Figure S3: valence band region and secondary electron emission cutoff region of ultraviolet photoemission spectroscopy (UPS) spectra for different EILs without and with a QD film deposited on top; Figure S4: derived energy level diagram from UPS measurement; Figure S5: characterization of control LED without QD film.
