# Supplementary material for: High-Performance Vertical Light-Emitting Transistors Based on ZnO Transistor/Quantum-Dot Light-Emitting Diode Integration and Electron Injection Layer Modification
Source: Micromachines (Basel). 2023 Oct 15;14(10):1933. doi: 10.3390/mi14101933 (PMC10609005; doi:10.3390/mi14101933)
Supplement: Supplementary file 1 [file micromachines-14-01933-s001.zip › micromachines-2648713-supplementary.pdf]

Supplementary Information

High-Performance Vertical Light-Emitting Transistors based on ZnO Transistor/Quantum-Dot Light-Emitting Diode Integration and Electron Injection Layer Modification

Jui-Fen Chang* and Jia-Min Yu

Department of Optics and Photonics, National Central University, Zhongli 320317, Taiwan

**jfchang@dop.ncu.edu.tw*

Transfer characteristics of ZnO transistor

The transfer characteristics of ZnO transistor were measured by alternately connecting the adjacent stripes of the interdigitated Ag electrodes on ZnO pattern as the source and drain contacts, corresponding to the channel length L and width W of 100 and 5000 μm, respectively. **Figure S1** shows the transfer characteristics performed at V_{ds}=5 V. The electron mobility of ZnO transistor at the saturation regime μ_{sat} can be calculated using the following equation:

$$\mu_{\text{sat}} = \frac{2L}{WC_{\text{i}}}\left(\frac{\partial\sqrt{I_{\text{ds}}}}{\partial V_{\text{gs}}}\right)$$

where C_i is the capacitance per unit area of the gate dielectric. By substituting L=100 μm, W=5000 μm, and C_i=350 nF/cm^2 for the 15 nm Al_2O_3/15 nm HfO_2 bilayer as the gate dielectric, μ_{sat} was calculated to be 10.4 cm^2/Vs.

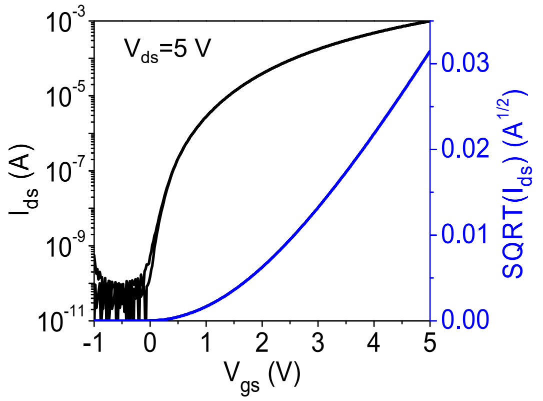

Figure S1. Transfer characteristics of ZnO transistor at V_{ds}=5 V.

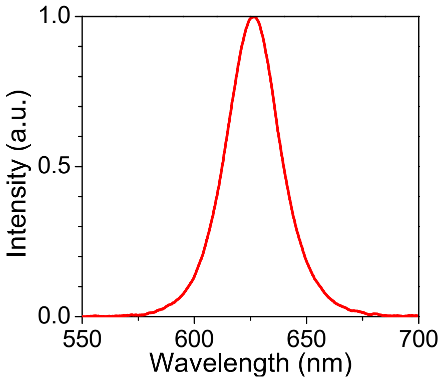

Figure S2. EL spectrum of QVLET without PEI in EIL.

Ultraviolet photoemission spectroscopy (UPS) measurement

We performed UPS measurements to evaluate the electron injection barrier from the electron injection layer (EIL) with different ZnO:PEI blend ratios into the CdSe/ZnS QD film. The samples were prepared under the same processing conditions on ITO glass as device fabrication. First, we measured a series of 25 nm EILs with different ZnO:PEI ratios, as the results shown in **Figure S3a,b**. For the EIL without PEI, i.e., pure ZnO film, we estimated the work function (WF) to be 5.23 eV and valence band (VB) to be 1.98 eV below the Fermi level (E_F), corresponding to an ionization potential of 7.21 eV. When PEI is blended in EIL, the effective WF seems to increase but does not show a systematic dependence on the PEI ratio. However, increasing the PEI ratio apparently leads to a lowered VB relative to E_F and corresponds to an increase in the ionization potential from 7.78 eV (PEI ratio of 0.17) to 8.29 eV (PEI ratio of 0.5), which may be correlated with the increased insulation of the blend film. Next, we measured a 25 nm CdSe/ZnS QD film deposited on EILs with different ZnO:PEI ratios, as the results shown in **Figure S3c,d**. Consistent with the result for bare EIL, increasing the PEI ratio in EIL was found to lower QD VB and enlarge ionization potential. Considering electron injection from ZnO in EIL into QD across the ZnS shell, and using a 3 eV bandgap of ZnO and a 3.6 eV bandgap of ZnS, we derived the energy level diagram for different samples, as shown in **Figure S4**. Based on the above results, we estimated that the injection barrier from PEI-free EIL to QD is only 0.09 eV and further decreases with increasing PEI. For the PEI ratios of 0.17 and above, the injection barrier has vanished, allowing energetically favorable injection.

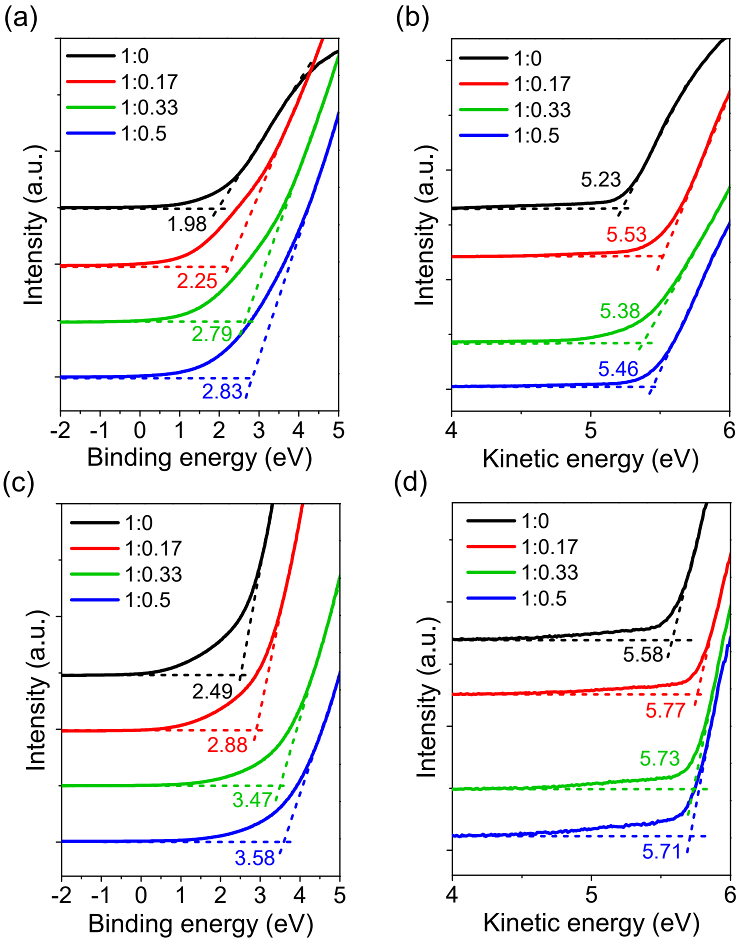

Figure S3. (a) VB region and (b) secondary electron emission cutoff region of UPS spectra for EILs with different ZnO:PEI ratios. (c) VB region and (d) secondary electron emission cutoff region of UPS spectra for a CdSe/ZnS QD film deposited on EILs with different ZnO:PEI ratios. The respective positions of VB and effective WF were extracted from the intersect of the linear extrapolation of the onset feature and the background level.

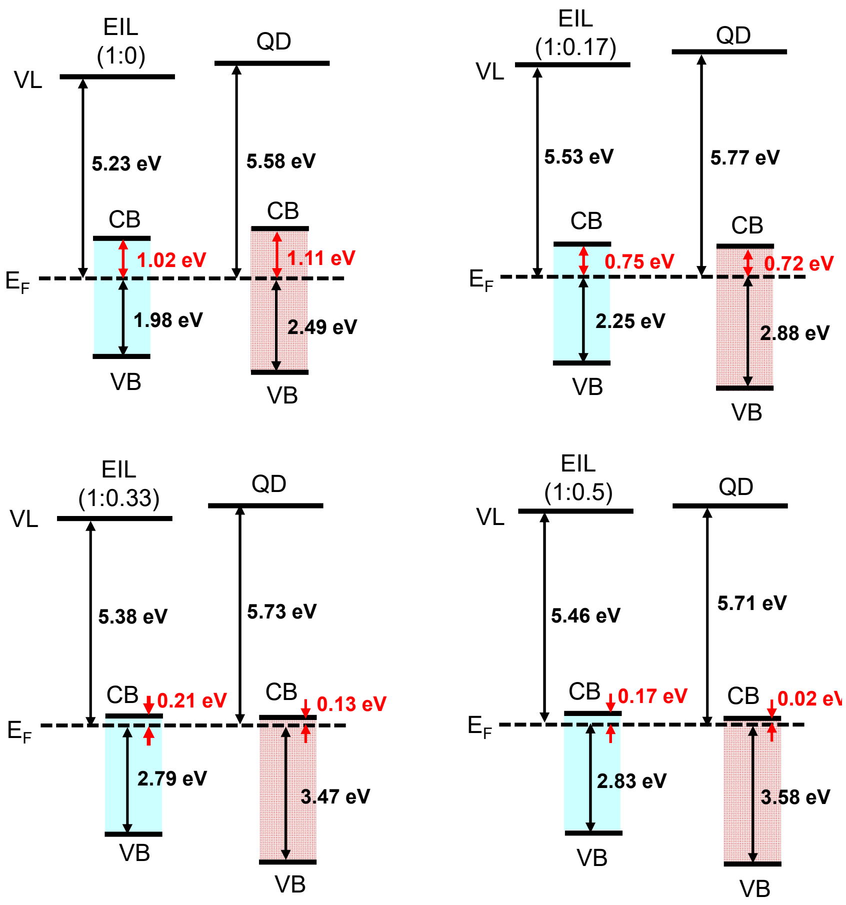

Figure S4. Derived energy level diagram of different EILs without and with a QD film deposited on top.

Characterization of control LED without QD film

In this work, we used the ZnO:PEI nanocomposite as EIL in the QVLET and QLED, and modified PEI ratio in EIL to optimize device performance. To understand whether PEI also facilitates electron injection from EIL into PVK/Poly-TPD layers, we characterized the control LED without QD film, as the device structure illustrated in **Figure S5a**. **Figure S5b-d** show the *J-V-L* characteristics, EQEs, and EL spectra of control LEDs with different ZnO:PEI ratios. No light emission is detected in the device without PEI. By contrast, blending PEI in EIL leads to a decreased current density but enhanced luminance and EQE. The highest EQE close to 10^{-2}% is achieved in the device with a PEI ratio of 0.33. This performance dependence on PEI ratio is similar to QVLET and QLED. The EL spectra mainly consist of two bands in 400-500 nm and 600-700 nm, which may stem from exciton emission in the Poly-TPD film bulk and exciplex emission at PVK/Poly-TPD interface, respectively. The emission from an ultrathin PVK layer is expected to be relatively weak.

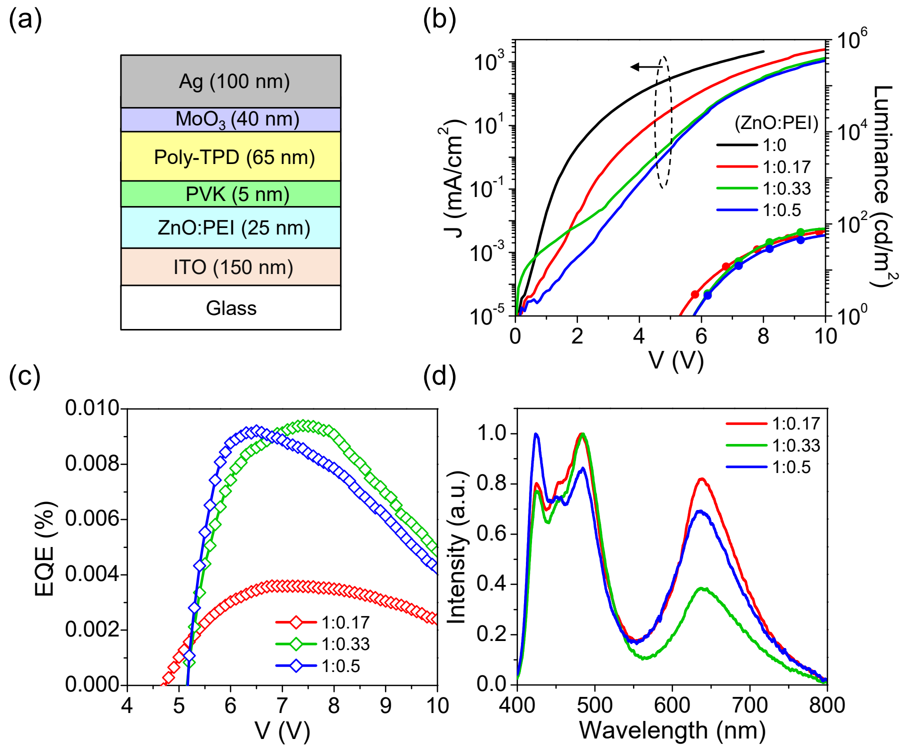

Figure S5. (a) Structure of control LED without QD film. (b) *J-V-L* characteristics, (c) EQEs, and (d) EL spectra of control LEDs with different ZnO:PEI ratios in EIL. No light emission is detected in the device without PEI.
